# Pushback: The Current Wave of Anti-Homosexuality Laws and Impacts on Health

**DOI:** 10.1371/journal.pmed.1001658

**Published:** 2014-06-24

**Authors:** Chris Beyrer

**Affiliations:** Center for Public Health and Human Rights, Johns Hopkins Bloomberg School of Public Health, Baltimore, Maryland, United States of America

## Abstract

Chris Beyrer discusses the worrying global trend towards the increasing criminalization of homosexuality.

*Please see later in the article for the Editors' Summary*

**Summary Points**

- The current period is one of rapid advances in Lesbian, Gay, Bisexual, and Transgender (LGBT) rights in many countries, and of a wave of anti-gay laws and policies in others.
- Gay, bisexual, and other men who have sex with men bear disproportionate burdens of HIV risk and face stigma and discrimination in accessing needed health services.
- The current wave of anti-gay laws and policies are likely to reduce access to health care, increase discrimination, and impact HIV research and programs.
- Close coordination with communities at risk will be key to program success in challenging contexts.

## Introduction

We are at an extraordinary moment in the struggle for Lesbian, Gay, Bisexual, and Transgender (LGBT) rights. Full citizenship rights, including those related to marriage equality, parenting, and health care are increasingly available to LGBT persons, couples, and families in countries as diverse as South Africa, Argentina, Spain, and the UK. 2013 was also a watershed year for LGBT rights in the US. Marriage equality advanced in a number of states and the landmark US Supreme Court decision United States v. Windsor ruled that much of the Defense of Marriage Act was unconstitutional—lifting the restrictions on full federal marriage rights for same-sex couples [1]. Though prejudice and discrimination against LGBT people remains a reality in all countries, the force of the law, and of the view that homosexuality is a normal variant of human sexuality, appear to have shifted decisively.

Globally, however, the situation remains markedly diverse, highly contentious, and the subject of vigorous debate in public fora, the media, and political life. While the European Union has mandated decriminalization of homosexuality as a requirement for membership, The Russian Federation enacted highly discriminatory legislation against homosexual “propaganda” in 2013, and used the EU stance on acceptance of homosexuality as a wedge issue to reduce support for European integration in Ukraine, and other states it seeks to influence [2]. The Indian Supreme Court in 2013 reversed a New Delhi High Court ruling that had struck down India's colonial era anti-sodomy laws, effectively re-criminalizing same-sex behavior between consenting adults [3]. The Delhi High Court decision had been advanced by India's National AIDS Control Organization, NACO, on the grounds that the sodomy statute was a barrier to HIV prevention services, and hence the decision was also widely seen as a setback for India's HIV response [4].

A wave of harsh and discriminatory legislation has further criminalized a range of activities in several African states, including Nigeria and Uganda, where homosexual relations were already illegal before the current legislative efforts began [5]. The Nigerian law, ostensibly put in place to ban same-sex marriage, includes a range of restrictions on freedom of assembly, speech, and association, which prompted UN High Commissioner for Human Rights, the Hon. Navi Pillay, to point out that the law violates human rights statutes and treaties to which Nigeria is signatory [6]. Pillay's comments during a recent official visit to Nigeria are telling:

“Another group living in fear is Nigeria's lesbian, gay, bisexual and transgender (LGBT) community. The new law known as the Same Sex Marriage (Prohibition Act) goes far beyond prohibiting same sex marriage – which was illegal anyway. The law violates international law in that it is discriminatory and seriously impinges on freedom of expression and freedom of assembly, and could lead to human rights defenders advocating for the rights of LGBT people receiving draconian prison sentences. There is also concern among medical specialists that it will have serious negative consequences for public health in Nigeria, by driving LGBT persons underground and deterring them from signing up for HIV educational programmes, prevention treatment and care services. Given that Nigeria currently has the second largest HIV epidemic in the world, this would be a heavy blow to the efforts to combat HIV...” [6].

Uganda's new law, which had been moving through its parliament in various versions for several years, was abruptly signed by President Yoweri Museveni on February 24, 2014 [7]. The law includes severe penalties for homosexual behavior, including life in prison for “aggravated homosexuality.” The law also makes failure to report known or suspected homosexual behavior a crime, greatly complicating efforts to work with LGBT populations, provide services, and address, among many other health issues, HIV, and other sexually transmitted infections (STIs). The implications for health care staff, and for research efforts and participants, are far-reaching. Similarly restrictive laws have been drafted or are under debate in 2014 in Kenya, DR Congo, Ghana, and Zimbabwe, among others, suggesting the current period of rights limitations is gaining rather than losing traction across Africa.

The similarities apparent in many of these laws, particularly their focus on criminalizing what they deem “homosexual propaganda,” is due, at least in part, to the reality that in several affected countries, notably Russia and Uganda, US evangelical activists and their organizations have been involved in developing these laws and lobbying for their passage [8],[9]. One such leader has been indicted in state courts in the US in a suit filed by Uganda LGBT activists alleging involvement in orchestrating their persecution [10]. This suit was upheld by a US federal judge and is ongoing.

## Implications for Health

What are the likely health consequences of these new laws and policies?

State-sponsored and community violence against known or suspected LGBT people is likely to increase [11]. There are already reports of brutal beatings and public humiliation in Russia, and in Uganda and Nigeria, several killings that have been attributed to vigilante violence against known or suspected sexual and gender minority persons. The recent torture and subsequent killings of prominent activists, including David Kato in Uganda and Eric Lembembe in Cameroon have gone uninvestigated, establishing dangerous precedents of impunity for crimes against LGBT persons [12].

Climates of fear are generated by these kinds of impunity, which force people to flee their communities, go into hiding, and avoid places, such as clinical or community outreach facilities, where they might be exposed to ridicule, violence, or arrest. Our research group reported on human rights violations against men who have sex with men (MSM) in Namibia, Botswana, and Malawi in 2007 [13]. In each country, men reported avoiding health care and HIV testing, due to fear of abuses or actual past experiences of mistreatment in health care facilities. Men who expressed such fears were less likely to have had a recent HIV test and more likely to have an untreated sexually transmitted infection [13].

In the case of Uganda or Nigeria it is perhaps too recent to be able to measure the health impacts of the new laws on LGTB populations, though there are data from prior to the new laws, since homosexuality was already illegal in both [6],[7]. The recent Ugandan police raid on a joint Makerere University–Walter Reed Army Institute for Research (WRAIR) Clinic in Kampala conducting HIV research among MSM and providing clinical services to this population suggests the impacts will be marked [14]. And we do have evidence from an earlier crackdown and wave of arrests in Senegal, which may shed light on how changes in policy and practice can dramatically reduce access to health care [15].

In Senegal, where same-sex behavior between consenting adults is illegal, considerable advances in provision of HIV services for MSM had been underway until 2008. In December 2008, just after Senegal had hosted the International Conference on AIDS in Africa (ICASA), a police crackdown led to the arrests and subsequent imprisonment of nine men who were all HIV prevention outreach workers for an MSM HIV program. The men were sentenced to 8 years in prison and substantial fines. They had been arrested and tried for “acts against nature” though no evidence was provided that any were found to be engaged in banned sexual activity. (The police had raided an NGO meeting after the conference.) A rapid qualitative assessment of the impacts of these arrests was conducted in 2009, some 6 months after the arrests, and showed immediate and marked declines in access to health care, fear of using services among MSM, and reports of men going into hiding. NGOs working with MSM suspended their activities, and providers reported sharp declines in MSM uptake and use of services [15].

In the Senegal case, appeals on behalf of the health care workers were successful, and all were released in 2009 because of insufficient evidence. Health care access has resumed, and Senegal, while its laws remain unchanged, demonstrates that health care can be provided even where same-sex relations remain criminalized. But the findings of the Senegal case are relevant for other states—arrests and other attacks on MSM communities can have marked impact on uptake and use of essential services.

## What Can Be Done?

All those engaged in efforts around universal access to health care, and with the gravity of the HIV epidemic among MSM in particular, are deeply concerned that the current wave of anti-gay legislation may have marked negative impacts on access and uptake of essential services. Research efforts too, are threatened. What approaches may help address these concerns?

In Uganda, the Civil Society Coalition on Human Rights and Constitutional Law (CSCHRCL) has recently released an enormously helpful set of suggested guidelines for international partners interesting in helping Ugandan efforts to repeal the anti-gay law, protect community members, and maintain access to health care [16]. An essential element of these requests is that the group *does not call* for any cuts in assistance to Uganda, including reductions in aid to programs like PEPFAR and the Global Fund. This is key, since it is never acceptable to trade off the rights of one group (LGBT individuals) against another (people living with HIV.) Activist calls to reduce aid to regimes that impose discriminatory laws may nevertheless be made, and it may become increasingly difficult to sustain assistance in the face of reports of brutality, abuse, and other rights violations [17].

The Uganda activists have called for international solidarity, and for support in their efforts to repeal the new law. They ask for those who support LGBT rights to speak publicly, to organize demonstrations, to reach out to multinational corporations with fair LGBT policies for employees and presence in Uganda to be proactive, and they request governments to consider asylum applications for LGBT persons fleeing persecution. It should be noted that US Secretary of State Kerry issued just such a directive to all US embassies and consulates in 2013.

Health research, including urgently needed HIV research for MSM, must continue. Such research efforts will likely become increasingly challenging in contexts where anti-homosexuality laws and practices become barriers to participant and staff safety. While research on populations who engage in activities like illicit drug use, sex work, and same-sex behavior have long been conducted world wide, risk-benefit ratios may shift where new laws and crackdowns come into play. Close and sustained coordination and communication with LGBT communities and leaders will be increasingly important in insuring protection of research subjects and staff. Countries with constitutional protections for sexual minorities, such as South Africa, may become more essential to the HIV research effort.

What else can be done? A primary principle is that the effort to address this global pushback against LGBT rights must be community led and locally focused. Activists on the ground know more about risks and protection, and about potential sympathizers and pressure points, than the best intentioned international partners. It is essential to avoid advocacy efforts (such as targeting health and development assistance) that communities do not support.

That said, the universality of human rights, and of the right of all persons to choose whom they love, must remain a fundamental part of international discourse and law. Without such rights and protections, we are all diminished.

## Abbreviations

MSM: men who have sex with men
